# Single-crystalline MAPbCl_3_ thin-films for photo- and X-ray voltaics

**DOI:** 10.1039/d5el00087d

**Published:** 2025-08-06

**Authors:** Waqas Zia, Mahdi Malekshahi Byranvand, Vishal Yeddu, Yuki Haruta, Dongyang Zhang, Makhsud I. Saidaminov, Michael Saliba

**Affiliations:** a Institute for Photovoltaics (ipv), University of Stuttgart 70569 Stuttgart Germany mahdi.malekshahi@ipv.uni-stuttgart.de michael.saliba@ipv.uni-stuttgart.de; b Helmholtz Young Investigator Group FRONTRUNNER, IMD-3 Photovoltaics, Forschungszentrum Jülich Jülich 52425 Germany; c Department of Chemistry, University of Victoria 3800 Finnerty Road Victoria British Columbia V8P 5C2 Canada msaidaminov@uvic.ca; d Department of Electrical & Computer Engineering, University of Victoria 3800 Finnerty Road Victoria British Columbia V8P 5C2 Canada

## Abstract

3 eV wide bandgap methylammonium lead trichloride (MAPbCl_3_) perovskites are promising for transparent solar cells, smart windows, and the internet of things (IoTs). However, it is challenging to crystallize uniform polycrystalline MAPbCl_3_ thin films from solution. On the other hand, single-crystalline MAPbCl_3_ can be grown as relatively uniform thin films. In this work, we demonstrate the fabrication of single-crystalline MAPbCl_3_ thin films on conductive glass substrates *via* a space-confined inverse temperature crystallization (ITC). The perovskite films exhibit no emission peaks from states located deep within the bandgap, confirming a less defective perovskite bulk than its polycrystalline counterpart. The resulting perovskite solar cells (PSCs) yield an open-circuit voltage (*V*_OC_) of up to 1.64 V and a high power conversion efficiency (PCE) of 1.1% under one sun irradiation. Additionally, the MAPbCl_3_ is studied for its conversion of X-rays into electrical energy, *i.e.*, “X-ray-voltaics”, demonstrating a *V*_OC_ of 0.89 V together with an output power of 3.57 μW cm^−2^ at an X-ray tube voltage of 60 kV (4900 μW cm^−2^).

## Abstract 2

Single-crystalline MAPbCl_3_ thin films grown *via* space-confined ITC enable transparent solar cells with a *V*_OC_ of 1.64 V and 1.1% PCE. They also advance X-ray voltaics, showing 0.89 V *V*_OC_ and 3.57 μW cm^−2^ output power under X-ray irradiation.

## Graphical abstract

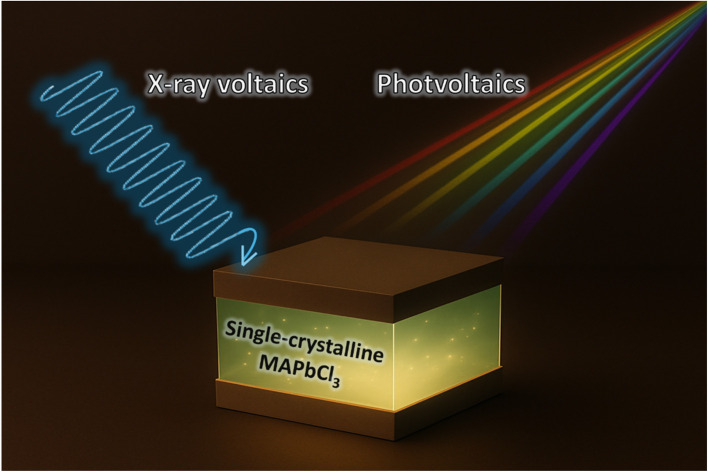

**Broader context**

While narrow-bandgap perovskites have been central to the development of high-efficiency solar cells, wide-bandgap counterparts, like MAPbCl_3_, are gaining interest. Its inherent transparency and the ability to yield high open-circuit voltages make it well-suited for applications like smart windows and advanced sensor systems, which can operate at very low current inputs. However, achieving a uniform polycrystalline MAPbCl_3_ defect-free thin film remains a significant challenge. We have demonstrated that growing single-crystalline thin films of MAPbCl_3_*via* inverse temperature crystallization results in highly crystalline thin films with minimal deep trap states, without requiring post-treatment. Apart from employing these single-crystalline MAPbCl_3_ thin films for the first time as solar cells, we also extend their use to X-ray voltaic devices. Notably, under X-ray exposure, wide-bandgap perovskites outperform their narrow-bandgap perovskites, an inverse trend compared to performance under solar illumination. This broadens the scope of MAPbCl_3_ beyond traditional photovoltaics, like industrial inspection, space radiation monitoring, and medical diagnostics. These diverse functionalities broaden the prospects of possible commercialization pathways of perovskites.

## Introduction

Metal-halide perovskite solar cells (PSCs) have attracted much attention in recent years due to their excellent optoelectronic properties^1–4^ based primarily on polycrystalline perovskite thin films. Despite their excellent progress in power conversion efficiencies (PCEs), the open-circuit voltage (*V*_OC_) deficit (compared to the bandgap) for wide bandgap perovskites can still be improved.^5–7^

Recently, we have reported, to our knowledge, the hitherto highest *V*_OC_ of 1.78 V for single-junction PSCs based on methylammonium lead chloride (MAPbCl_3_).^8^ However, considering the wide bandgap (*E*_g_) of 3.03 eV for MAPbCl_3_, there is still a significant loss-in-potential (*i.e.*, *E*_g_/*q* − *V*_OC_), since the potential *V*_OC_ for MAPbCl_3_ is 2.69 V (corresponding to a loss-in-potential of 0.34 V).^9^ Our work has shown that the crystallization of MAPbCl_3_ thin films with a homogenous morphology and a uniform interface is quite challenging because of the lower solubility of chloride salts in organic solvents and fast crystallization kinetics. This results in excessive non-radiative recombination, leading to a considerable loss-in-potential.^8^

However, the solution-grown cubic and transparent bulk MAPbCl_3_ single crystals have been used for photodetector applications.^10,11^ On a similar basis, PSCs based on single-crystalline films have been demonstrated using various perovskites, *e.g.*, MAPbI_3_ (1.55 eV), MAPbBr_3_ (2.3 eV), FA_0.6_MA_0.4_PbI_3_ (1.48 eV), and FAPbI_3_ (1.5 eV).^12–15^ However, in contrast to bulk perovskite single crystals, which are many centimeters in size, growing single-crystalline films on the substrate is challenging because of unwanted nucleation sites, control of thickness, and growth dynamics. One method for answering these challenges is the space-confined inverse temperature crystallization (ITC) method that has been used for single-crystalline thin films.^16,17^

Additionaly, these single-crystalline thin films, due to their relatively high thicknesses, can also absorb more effectively high-energy neutrons, alpha (α) particles, beta (β) particles, and X-rays into electrical energy.^18,19^ Zhao and co-workers have demonstrated β-voltaics based on polycrystalline MAPbBr_3_ and mixed-cation PSCs. These β-voltaic cells find their applications in space electronics and implantable medical devices due to their long lifespan, high energy density, and durability.^20,21^ In contrast, X-rays offer better energy conversion owing to their higher penetration depth than β-particles. For example, MAPbI_3_ single-crystalline solar cells have recently been tested as X-ray voltaic devices.^22^

A sufficiently thick perovskite film with a wide bandgap is needed to absorb high-energy particles, enhance their energy deposition, and minimize thermal losses. Polycrystalline perovskite thin films are typically limited to ∼1 μm. On the other hand, single-crystalline perovskite films are free of grain boundaries, have high charge mobilities, and are relatively thick.^23–26^ Here we focus on single-crystalline MAPbCl_3_ with a wide bandgap that reduces the thermal losses from high-energy particles. In addition, single-crystalline MAPbCl_3_ films can be crystallized without annealing or post-treatment steps to improve the film morphology. These single-crystalline MAPbCl_3_ films can be integrated into X-ray voltaic cells to absorb high-energy X-rays, suitable for applications like security screening, flaw detection, and environmental monitoring.^27^

Here, among the first time, we fabricate single-crystalline MAPbCl_3_ thin films *via* the space-confined ITC method for both photovoltaic and X-ray voltaic applications. The devices, without any passivation or post-treatment steps, show *V*_OC_'s of up to 1.64 V with one of the highest PCEs of 1.1%, under one sun illumination. Despite high crystalline quality and absence of grain boundaries, a higher *V*_OC_ compared to passivated polycrystalline thin films (a few hundred nm's thick) has not been achieved. This can be related to the higher thickness of single-crystalline films (a few μm's thick), which may result in a higher saturation current density due to an increased non-radiative recombination processes. In addition, the diffusion length may not scale according to the layer thickness further inhibiting the *V*_OC_. Solar cells using this material are further employed as X-ray voltaic cells. At an X-ray tube voltage of 60 kV (equal to an input power density of 4900 μW cm^−2^), a *V*_OC_ of 0.89 V is obtained along with an output power density of 3.57 μW cm^−2^. According to the best of our knowledge, this is the highest power conversion achieved by X-rays for PSCs. These initial results show that by carefully optimizing the crystallization process and thickness of MAPbCl_3_, an alternative use case can be established for widest bandgap materials.

## Results and discussion

**Fig. 1:**
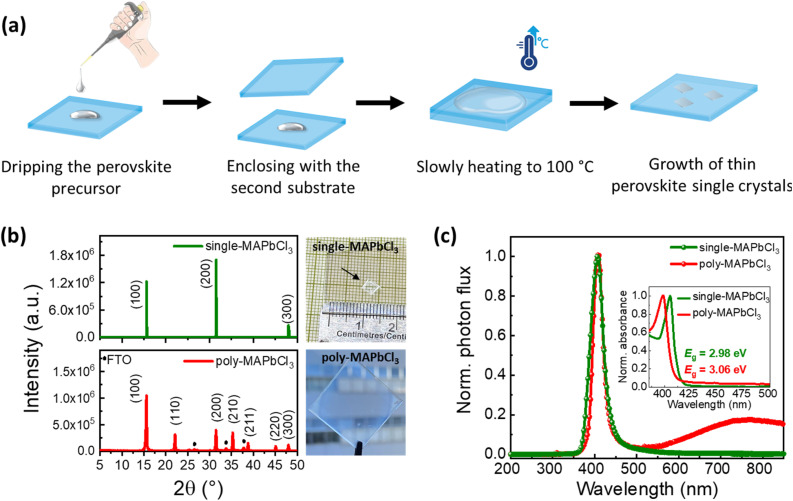
(a) Schematics showing the steps in fabricating single-crystalline MAPbCl_3_ solar cells *via* space-confined ITC. (b) XRD spectra of the single-crystalline and polycrystalline MAPbCl_3_ thin films. (c) Normalized PL spectrum of the single-crystalline and polycrystalline thin films measured at an excitation wavelength of 343 nm (inset: normalized absorbance spectra showing a bandgap of 2.98 eV and 3.06 eV for the single-crystalline and polycrystalline MAPbCl_3_ thin films respectively (extracted Tauc plots in Fig. S1 in the SI).

Single-crystalline MAPbCl_3_ thin films are grown by the space-confined ITC method.^16,17^ The experimental details can be found in the SI. Briefly, the perovskite precursor solution is sandwiched between two substrates, and the temperature is increased slowly to 100 °C (Fig. 1a). When the solution reaches the supersaturation limit, a single-crystalline thin film grows within the confined space.

We note that single-crystalline MAPbCl_3_ films form without any additional annealing or passivation step at ambient conditions in air and are highly crystalline. This is confirmed by the crystallographic properties of the obtained MAPbCl_3_ films *via* X-ray diffraction (XRD), showing intense reflections at (100), (200), and (300) planes at diffraction angles of 15.59°, 31.50°, and 47.98°, respectively. There is no additional diffraction response from other planes (Fig. 1b). When compared to our previously reported polycrystalline MAPbCl_3_ thin films,^15^ additional multiple reflections from different planes can be observed at 22.12°, 35.32°, and 38.78°.

For the optical properties, photoluminescence (PL) measurements for both single- and polycrystalline thin films are performed. Fig. 1c shows the band-to-band radiative recombination peaks at 406 nm in both cases. For the polycrystalline thin film, an additional peak can be observed at 750 nm, indicating a band-to-defect recombination peak. Consistent with our previous findings, this peak confirms the existence of deep defect states in the polycrystalline MAPbCl_3_ perovskite.^8^

On the other hand, this additional peak has not been observed for the single-crystalline sample, which is consistent with the reduction of deep traps in the bulk. However, the asymmetry of the PL spectrum for single-crystalline MAPbCl_3_ has already been reported in the literature and is attributed to the presence of Cl^−^ vacancies at the crystal surface.^11^ This highlights that MAPbCl_3_ appears to be intrinsically more prone to surface defects due to the lower formation energy (−0.7 eV) in chlorine compared to iodine (−0.1 eV) and bromine (0.25 eV)-based perovskites.^28^

The UV-vis absorption spectra of single- and polycrystalline thin films are illustrated in Fig. 1c (inset). The extracted Tauc plot of single-crystalline thin film shows a slightly lower bandgap of 2.98 eV compared to polycrystalline thin film of 3.06 eV (see Fig. S1 in the SI), which follows the reported bandgap values for single-^29^ and polycrystalline^30^ MAPbCl_3_ thin films. The narrowing of the bandgap for perovskite single crystals due to their thickness-dependent below-bandgap absorption is well reported in the literature and attributed to the slight transition of direct bandgap to indirect bandgap.^31–33^ It has been argued that, for thicker perovskite films, this transition occurs due to the distortion of the lead iodide framework. This results in an electric field across the Pb atom and, hence, splits the conduction band *via* Rashba splitting.^34^

**Fig. 2:**
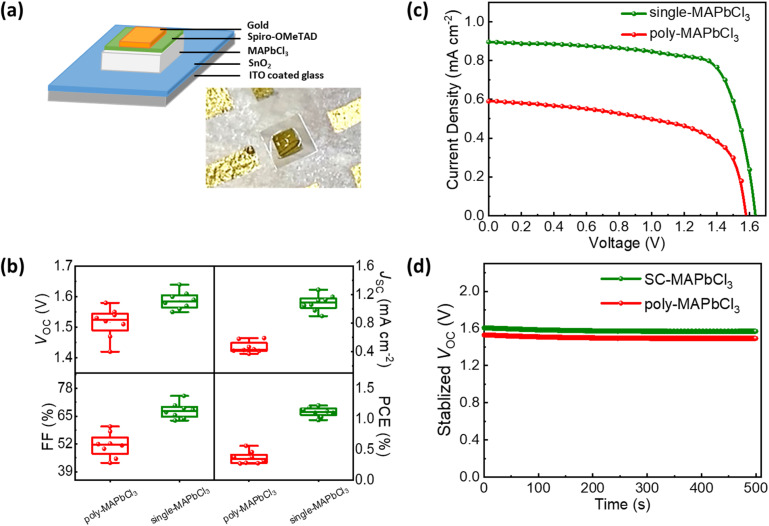
(a) Layer stack of a single-crystalline MAPbCl_3_ solar cell with a n-i-p configuration, together with a photograph of a single-crystalline MAPbCl_3_ solar cell (based on a single crystal of size 2.3 mm × 2.3 mm). (b) Device statistics for *V*_OC_, *J*_SC_, FF, and PCE of single-crystalline and polycrystalline MAPbCl_3_ solar cells (*n* = 8 devices). (c) *JV*-curves of the champion solar cells. (d) Stabilized *V*_OC_ for 500 s for both single- and polycrystalline MAPbCl_3_ solar cells.

In the next step, we incorporate the single-crystalline films into a solar cell stack of glass/ITO/SnO_2_/MAPbCl_3_/Spiro-OMeTAD/Au. Fig. 2a shows a photograph of a single-crystalline MAPbCl_3_ solar cell (see the Experimental section in SI). On the other hand, polycrystalline MAPbCl_3_ solar cells are fabricated using a layer stack glass/FTO/c-TiO_2_/mp-TiO_2_/MAPbCl_3_/Spiro-OMeTAD/Au as described in our previous work.^8^ We note that due to the poor film coverage of polycrystalline MAPbCl_3_ films, planar SnO_2_ electron transport layer (ETL) frequently leads to shunting paths, resulting in non-functional devices, as shown in Fig. S2. Owing to this, a mesoporous TiO_2_-based ETL is used to avoid shunting, as its mesoporous structure embeds the perovskite efficiently. However, the same mesoporous TiO_2_ layer might not provide a smooth surface for the growth of single-crystalline thin films, as the underlying mesoporous film would induce unwanted heterogeneous nucleation sites, hampering the formation of single crystals.^35^ Hence, a facile, planar SnO_2_ ETL on an ITO substrate is needed to achieve high-quality single-crystalline MAPbCl_3_ films.

Fig. 2b shows the statistical data of the photovoltaic parameters for both single- and polycrystalline solar cells with 8 devices in each case under one sun. The single-crystalline devices show remarkable reproducibility with an average *V*_OC_ = 1.59 ± 0.03 V, short-circuit current density (*J*_SC_) = 1.09 ± 0.11 mA cm^−2^, a fill factor (FF) = 67.6 ± 3.66% and a PCE = 1.11 ± 0.08%. On the other hand, polycrystalline devices show an average *V*_OC_ of 1.52 ± 0.05 V, *J*_SC_ = 0.46 ± 0.08 mA cm^−2^, FF = 51.5 ± 5.73%, and PCE = 0.36 ± 0.10%.

The champion single-crystalline MAbCl_3_ solar cell results in a *V*_OC_ of 1.64 V, *J*_SC_ of 0.9 mA cm^−2^ and a FF of 74.5% with a PCE of 1.10%. To the best of our knowledge, this represents the highest reported PCE for MAPbCl_3_ solar cells. On the other hand, the champion polycrystalline MAPbCl_3_ solar cell has yielded a lower *V*_OC_ of 1.58 V, *J*_SC_ of 0.59 mA cm^−2^, and a FF of 60.2%, leading to a significantly lower PCE of 0.56% (see Fig. 2c, Table 1). Since *V*_OC_ is the main performance criteria for these wide bandgap perovskites with significantly lower currents, we perform long-term *V*_OC_ tracking on these devices. Fig. 2d shows stabilized *V*_OC_ for 500 s under constant illumination for both single- and polycrystalline MAPbCl_3_ thin films, highlighting their significant long-term stability.

**Table 1:** Photovoltaic parameters of single- and polycrystalline MAPbCl_3_ solar cells

	*V*_OC_ (V)	*J*_SC_ (mA cm^−2^)	FF (%)	PCE (%)
poly-MAPbCl_3_	1.58	0.59	60.2	0.56
single-MAPbCl_3_	1.64	0.90	74.5	1.10

Although the single-crystalline MAPbCl_3_ thin films grown by the space-confined ITC method are of high quality in terms of their crystallinity and optical properties, still, a *V*_OC_ of 1.64 V is lower than the theoretical limit of 2.69 V for a *E*_g_ of ∼3.1 eV, as reported by Lunt *et al.*^9^

**Fig. 3:**
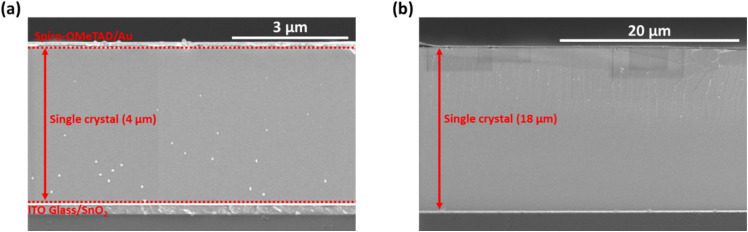
SEM cross-section of a single-crystalline MAPbCl_3_ thin film solar cell (a) with a thickness of ∼4 μm (b) with a thickness of ∼18 μm.

In our previous work on polycrystalline MAPbCl_3_ thin films, annealing of the films in MACl vapor atmosphere has resulted into an improved film morphology, leading to a *V*_OC_ of 1.78 V.^8^ Using such post-treatments in various annealing atmospheres here for a prolonged annealing at high temperatures result in the detachment of the single crystals, leading to no electrical contact between MAPbCl_3_ and the SnO_2_ ETL. In addition, one of the main reasons for the lower *V*_OC_ is the higher thickness of single-crystalline thin films, as polycrystalline perovskite thin films grown by spin-coating are typically a few hundred nanometers thick, whereas single-crystalline thin films are usually a few μm's thick. Due to the diffusion length not necessarily scaling with thickness, even an improved bulk quality may preclude charges from diffusing all the way to their (now far away) respective contacts. Fig. 3(a) and (b) show the cross-section scanning electron microscope (SEM) images of two different single-crystalline MAPbCl_3_ thin film solar cells consisting of ITO/SnO_2_/MAPbCl_3_/spiro-OMeTAD/Au layer stack. These images show that the thickness of single-crystalline MAPbCl_3_ thin films ranges from 4 μm to 18 μm. The diffusion of charge carriers toward the surface of a semiconductor becomes less efficient if the surface lies further away.^36^

This can be further explained with the help of the diode equation for a single junction solar cell 
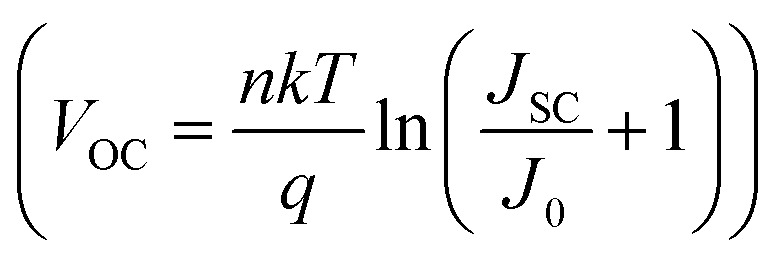
, where *n* is the ideality factor, *k* is Boltzmann constant, *T* is the temperature, and *J*_SC_, *J*_0_ are the photogenerated and saturation current densities, respectively. The increase in the saturation current density *J*_0_ is generally correlated with the increase in non-radiative recombination in a solar cell and, hence, a decrease in the *V*_OC_.^37,38^ Kirchartz and co-workers have reported that *J*_0_ within the bulk increases linearly with the increase in the absorber layer thickness. With a thicker perovskite layer, more photons can be absorbed, and hence, more photogenerated charge carriers are produced. However, at the same time, it triggers more non-radiative recombination mechanisms within the perovskite bulk, which leads to significant *V*_OC_ losses.^39,40^ Huang and his team have shown a decrease in the *V*_OC_ of single-crystalline MAPbI_3_ solar cells with an increase in the thickness of single-crystalline thin films up to 40 μm.^31^ Similarly, Turedi *et al.* demonstrated that increasing the thickness of the perovskite layer in single-crystalline solar cells led to a decline in device performance, attributed to inefficient charge carrier collection.^41^

One more aspect of this lower *V*_OC_ is the narrowed bandgap of the perovskite single crystals from 3.03 eV to 2.98 eV. In addition, the fabrication process of thin film single-crystalline solar cells at a μm scale is not yet completely optimized and has its own challenges, such as varying space between the two sandwiched substrates. This leads to a difference in thickness between various single crystals grown on a single substrate. Optimization could entail constant space between the substrates and increasing the amount of precursor between substrates to ensure larger crystals.^42^ Furthermore, the thickness of as-grown single-crystalline thin films can be reduced by laser polishing as shown by Kedia *et al.*^43^ This would result in thin single-crystalline layers of uniform thickness with a much smoother interface with the HTL. On the other hand, an increased volume of precursor solution with a lower concentration can help to avoid fast supersaturation and, hence, inhibit the multiple nucleation sites. In addition to that, careful post-treatments need to be defined that may help to improve the surface morphology of these single-crystalline thin films without detaching them from the substrates.

Finally, these devices, as a proof of concept, are further used as X-ray voltaic cells at different X-ray tube voltages. The performance of X-ray voltaic cells is governed by the maximum amount of energy deposited in the perovskite layer. Different factors influence the energy dosage of X-rays in the perovskite layer. The accelerating voltage of the incoming electrons in the X-ray tube and the thickness of the layers lying above the perovskite are very critical. If the accelerating voltage is too low, then most of the energy will be deposited into the front layers before the X-rays reach the perovskite layer. Similarly, if the accelerating voltage is too high, the X-rays will simply penetrate through the perovskite layer, and the according energy would be wasted. Keeping that in mind, in this work, the single-crystalline MAPbCl_3_ devices have been exposed to X-rays from the Au contact side instead of from the glass side to avoid the energy deposition of X-rays in the thick glass substrate rather than in the perovskite layer.^20^

**Fig. 4:**
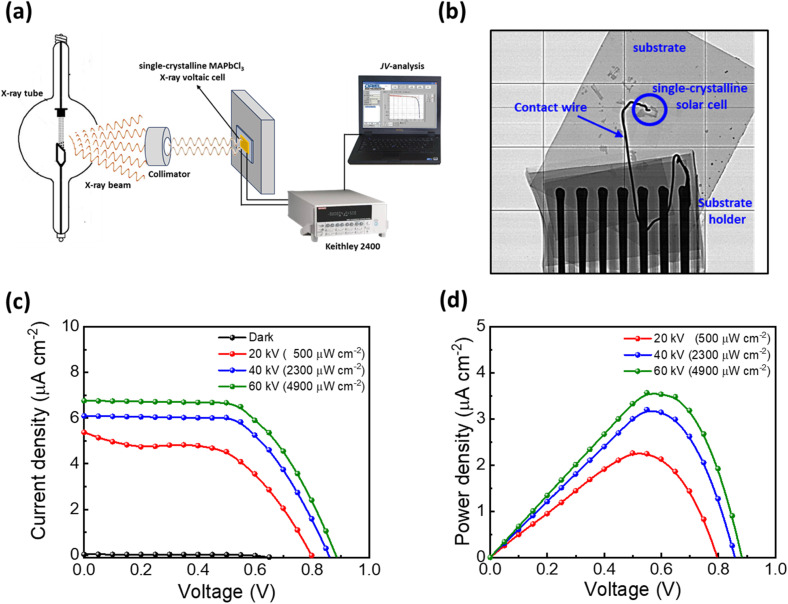
(a) Schematics of the X-ray voltaic setup consisting of a tungsten-based X-ray tube (b) X-ray image of a single-crystalline MAPbCl_3_ solar cell on an ITO substrate contacted with a contact wire to measure IV-traces. (c) *JV*-curves of the champion single-crystalline MAPbCl_3_ X-ray voltaic cells at different X-ray tube voltages corresponding to different power densities (d) Output power curves of single-crystalline MAPbCl_3_ X-ray voltaic cells at different X-ray tube voltages.

Since the energy of X-rays is significantly higher than the bandgap of the perovskites, MAPbCl_3,_ with its wide bandgap, can be used for X-ray voltaics. The higher energy of the X-rays results in the formation of hot carriers with high kinetic energy, which equilibrate themselves by dissipating the heat energy to the lattice instead of taking part in the power generation. Hence, I^−^ and Br^−^-based narrow bandgap perovskites are (relatively) less efficient for this purpose.^21^ Here, MAPbCl_3_ single-crystalline devices are measured as X-ray voltaic cells using a tungsten-based X-ray tube. Polycrystalline-MAPbCl_3_ thin films cannot be employed for X-ray voltaics as their thickness is too low for X-rays to deposit any energy, resulting in no current signal. The X-ray beam is collimated using a 10 mm diameter aluminum slit and is directed on the X-ray voltaic cell. The complete schematic of the setup is shown in Fig. 4a. An X-ray image of the X-ray voltaic cell is taken to make sure that the device lies within the focus of the X-ray beam, as shown in Fig. 4b. The X-ray tube voltage is increased from 20 kV to 60 kV at a constant tube current of 1 mA to observe the influence of the tube voltage on the device performance. The corresponding power densities at each tube voltage (calculated *via* SpekPy^44^ and counter checked by Monte Carlo Simulations^45^) range from 500 μWcm^−2^ to 4900 μW cm^−2^. Currently, our setup is restricted from going beyond 60 kV as it can cause adverse effects on the X-ray tube life.

With increasing the tube voltage, the single-crystalline devices have shown improved X-ray voltaic parameters, as shown in Fig. 4c: at a tube voltage of 20 kV equal to an input power density of 500 μW cm^−2^, a *J*_SC_ of 5.40 μA cm^−2^, along with a *V*_OC_ of 0.8 V, leading to an output power density of 2.26 μW cm^−2^. For 40 kV (2300 μW cm^−2^), *J*_SC_ and *V*_OC_ have increased to a value of 6.08 μA cm^−2^ and 0.86 V, respectively, with a power conversion of 3.2 μW cm^−2^. The highest *V*_OC_ of 0.89 V with a *J*_SC_ of 6.73 μA cm^−2^ is obtained at a tube voltage of 60 kV (4900 μW cm^−2^) with an output power density of 3.57 μW cm^−2^. The output power curves corresponding to different X-ray input powers have been shown in Fig. 4d. To the best of our knowledge, this is the highest output power achieved by perovskite X-ray voltaic cells. Previously, Moazzezi *et al.* have reported an output power of 0.33 μW cm^−2^ for single-crystalline MAPbI_3_-based X-ray voltaic cells.^22^ In a similar approach, an output power of ∼0.14 μW has been reported for AlGaInP based X-ray voltaics.^46^ Our findings further strengthen that wide bandgap perovskites exhibit significantly superior performance compared to narrow bandgap ones, when exposed to radiant sources like X-rays.^21^ This is contrary to their trend under one sun condition, where photovoltaic power conversion decreases with the increase in bandgap. This efficient harvesting of radiant energy shows the potential of MAPbCl_3_ to meet the needs of low-power devices such as micro-electro-mechanical systems (MEMS) during space missions. These self-powered X-ray voltaic devices may also eliminate the need for a battery to power the devices.^46^

Moreover, compared to MAPbI_3_ and MAPbBr_3_, MAPbCl_3_ exhibits superior intrinsic stability due to its more negative enthalpy of formation,^47,48^ stronger Pb–Cl bonds that inhibit defect formation and degradation,^49,50^ and enhanced resistance to moisture.^51^ These characteristics make it particularly well-suited for the environments and applications discussed above. At the same time, there is still significant scope for improving the device's performance under X-ray irradiation. For example, the thickness of perovskite, ETL, HTL, and metallic thin films could be further optimized such that the maximum energy deposition occurs in the absorber layer. Furthermore, standardized measurement conditions for X-ray voltaics are also very crucial for enhancing their performance.

## Conclusion

In conclusion, we demonstrate the potential of MAPbCl_3_ perovskite in the form of single-crystalline thin films for both photo and X-ray voltaics. Our results show that, compared to polycrystalline thin films, high-quality single-crystalline MAPbCl_3_ thin films can be crystallized out without any annealing or passive step. These single-crystalline thin films result in a *V*_OC_ of 1.64 V and a PCE of 1.10%. At the same time, currently, the increased thickness of single-crystalline thin films results in enhanced non-radiative recombination processes affecting the overall diffusion of the charge carriers, leading to a relatively lower *V*_OC_. As a proof of concept, these single-crystalline MAPbCl_3_ thin films are further employed as X-ray voltaic cells. At a tube voltage of 60 kV, this leads to a *V*_OC_ of 0.89 V and a remarkable output power of 3.57 μW cm^−2^, highlighting the potential of the widest bandgap perovskites for novel use cases beyond solar applications.

## Conflicts of interest

There are no conflicts to declare.

## Supplementary Material

EL-001-D5EL00087D-s001

## Data Availability

The data will be available upon reasonable request. Experimental details, characterization methods, Tauc plots, and *JV*-curve of a planar shunted polycrystalline solar cell are available in the supplementary information. See DOI: https://doi.org/10.1039/d5el00087d.
