# Supplementary material for: Single-crystalline MAPbCl3 thin-films for photo- and X-ray voltaics
Source: EES Solar. 2025 Aug 6;1(5):810–8. doi: 10.1039/d5el00087d (PMC12355366; doi:10.1039/d5el00087d)
Supplement: EL-001-D5EL00087D-s001 [file EL-001-D5EL00087D-s001.pdf]

Supplementary Information (SI) for EES Solar.
This journal is © The Royal Society of Chemistry 2025

Single-Crystalline $MAPbCl_3$ Thin-Films for Photo- and X-ray Voltaics

Waqas Zia,[a,b] Mahdi Malekshahi Byranvand,[a*] Vishal Yedu,[c] Yuki Haruta,[c] Dongyang Zhang,[c] Makhsud I. Saidaminov,[c,d*] and Michael Saliba [a,b*]

[a]*Institute for Photovoltaics (ipv), University of Stuttgart, 70569 Stuttgart, Germany*

[b] *Helmholtz Young Investigator Group FRONTRUNNER, IMD-3 Photovoltaics, Forschungszentrum Jülich, 52425 Jülich, Germany*

[c]*Department of Chemistry, University of Victoria, 3800 Finnerty Road, Victoria, British Columbia V8P 5C2, Canada.*

[d]*Department of Electrical & Computer Engineering, University of Victoria, 3800 Finnerty Road, Victoria, British Columbia V8P 5C2, Canada.*

Experimental

Materials

N,N-dimethylformamide (DMF, 99.8%), dimethylsulfoxide (DMSO, 99.9%), titanium diisopropoxide bis(acetylacetonate), Spiro-OMeTAD, 4-tert-butylpyridine (TBP), chlorobenzene (CB), and lithium bis (trifluoromethylsulfonyl) imide salt (Li-TFSI) were purchased from Sigma Aldrich Co., Ltd. Lead (II) chloride ($PbCl_2$, 99.999%) was purchased from TCI Chemicals. Methylammonium chloride (MACl, 99.9%) was purchased from GreatCell Solars. TiO_2 30 nm particle paste (30 NR-D) was purchased from Dyesol. SnO_2 colloid precursor (tin (IV) oxide, 15% in H_2O colloidal dispersion) was purchased from Alfa Aesar. All salts and solvents were used as received without any further purifications.

Fabrication of single-and polycrystalline $MAPbCl_3$ thin film solar cells

The pre-patterned 75 mm × 75 mm indium tin oxide (ITO) coated glass substrates (sheet resistance 8-9 Ω sq-1, Luminescence Technology) were cleaned consecutively with 2% Hellmanex water solution, deionized (DI) water, acetone, and isopropyl alcohol in an ultrasonic bath for 15 min, followed by UV-Ozone treatment for 15 min. After drying, the substrates were cleaned with ultraviolet ozone (UVO) for 15 minutes. Next, the SnO_2 solution (1 mL of SnO_2 colloidal precursor in 3 mL of deionized water) was spin-coated on the cleaned ITO substrates at 3000 rpm for 30 s, followed by annealing at 150 °C for 30 minutes. The SnO_2 films were further cleaned with UVO treatment for 20 minutes. After that, the single-crystalline perovskite thin films were grown by using the space-confined inverse temperature crystallization as reported in [1,2]. These substrates

were placed on a heating plate at 25 °C and a 150 μL precursor solution of 1M $MAPbCl_3$ in DMF/DMSO (1:1) was dropped onto the substrates. After that, each substrate with the solution on it was enclosed by another SnO_2-coated substrate. The temperature of the heating plate was increased from 25 °C to 40 °C at a rate of 15 °C h^{-1} and then the temperature was further increased to 100 °C at a rate of 4 °C h^{-1}. The substrates were kept at 100 °C for 5 h. After that, the substrates were cooled down slowly and then separated using a blade. The excess of the solvent was dried using a wipe. Next to that, the Spiro-OMeTAD solution was deposited on these single-crystalline $MAPbCl_3$ thin films using the procedure explained above. After that, each single crystal was masked using a Kapton tape and then 80 nm gold electrodes were thermally evaporated on all the crystals. The active area of the cells was defined by black tape.

Polycrystalline $MAPbCl_3$ solar cells were fabricated using the method described in our previous work.[3]

Characterization

The field emission SEM (HITACHI,) was used to characterize the morphologies of the films with an accelerating voltage of 3 kV. The UV-Vis absorbance spectra of the perovskite films were measured with a PerkinElmer LAMBDA 0101. The photoluminescence (PL) spectra were measured using a Carl Zeiss LSM800 confocal microscope. The perovskite film crystallinity was examined by an X-ray diffraction (XRD, SmartLab SE from Rigaku) machine with a HyPix-400 (2D HPAD) detector. The solar cells were characterized using a 21-channel LED Solar Simulator (Wavelabs Solar Metrology Systems) with an AM 1.5G spectrum (100 mW cm^{-2}). The J–V characteristics were measured in both reverse and forward directions with a constant scan rate of circa 50 mVs-1 (Keithley 2400 source measurement unit).

For X-ray voltaic cells, A Comet MXR-160/22 X-ray tube with a tungsten anode was used as an X-ray source. The tube voltage was set to 40 keV and the current was set to 1 mA. Keithley 2450 was used to measure current and voltage.

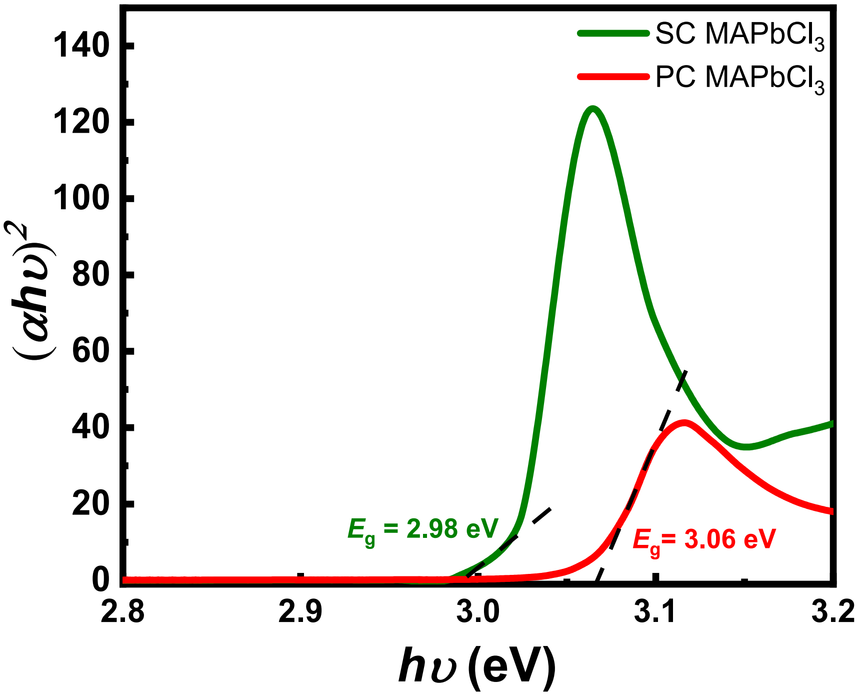

Figure S1 Tauc plots showing a bandgap of 2.98 eV and 3.06 eV for single-crystalline and polycrystalline $MAPbCl_3$ thin films respectively.

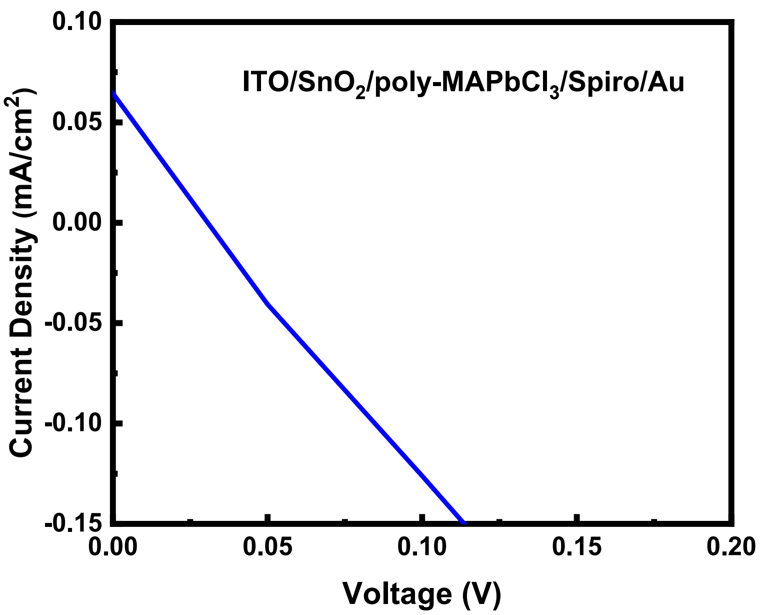

Figure S2 J-V Curve of a shunted polycrystalline $MAPbCl_3$ solar cell fabricated on a planar SnO_2 ETL layer."

References

(1) Chen, Z.; Dong, Q.; Liu, Y.; Bao, C.; Fang, Y.; Lin, Y.; Tang, S.; Wang, Q.; Xiao, X.; Bai, Y.; Deng, Y.; Huang, J. Thin Single Crystal Perovskite Solar Cells to Harvest Below-Bandgap Light Absorption. Nat Commun 2017, 8 (1), 1890. https://doi.org/10.1038/s41467-017-02039-5.

(2) Turedi, B.; Lintangpradipto, M. N.; Sandberg, O. J.; Yazmaciyan, A.; Matt, G. J.; Alsalloum, A. Y.; Almasabi, K.; Sakhatskyi, K.; Yakunin, S.; Zheng, X.; Naphade, R.; Nematulloev, S.; Yeddu, V.; Baran, D.; Armin, A.; Saidaminov, M. I.; Kovalenko, M. V.; Mohammed, O. F.; Bakr, O. M. Single-Crystal Perovskite Solar Cells Exhibit Close to Half A Millimeter Electron-Diffusion Length. Advanced Materials 2022, 34 (47), 2202390. https://doi.org/10.1002/adma.202202390.

(3) Zia, W.; Malekshahi Byranvand, M.; Rudolph, T.; Rai, M.; Kot, M.; Das, C.; Kedia, M.; Zohdi, M.; Zuo, W.; Yeddu, V.; Saidaminov, M. I.; Flege, J. I.; Kirchartz, T.; Saliba, M. MAPbCl3 Light Absorber for Highest Voltage Perovskite Solar Cells. ACS Energy Lett. 2024, 9 (3), 1017–1024. https://doi.org/10.1021/acsenergylett.3c02777.
